# Retraction Note: Metal free cross-dehydrogenative N-N coupling of primary amides with Lewis basic amines

**DOI:** 10.1038/s41467-024-49357-z

**Published:** 2024-06-18

**Authors:** Subban Kathiravan, Prakriti Dhillon, Tianshu Zhang, Ian A. Nicholls

**Affiliations:** 1 Bioorganic & Biophysical Chemistry Laboratory, Centre for Biomaterials Chemistry, Department of Chemistry & Biomedical Sciences, Linnaeus University, Kalmar, SE-39182 Sweden; 2 Attana AB, Greta Arwidssons väg 21, 11419 Stockholm, Sweden

**Keywords:** Synthetic chemistry methodology, Catalyst synthesis, Synthetic chemistry methodology

Retraction to: *Nature Communications* 10.1038/s41467-024-46890-9, published online 26 March 2024

The authors have retracted this article. After publication, they were made aware of an error in the characterization of the hydrazide products reported. The results and conclusions presented are therefore incorrect. All authors agree with this retraction.

